# Case Report: Rare intraventricular H3 K27-altered diffuse midline glioma in an adult

**DOI:** 10.3389/fonc.2025.1477978

**Published:** 2025-03-20

**Authors:** Merari Jasso, Jay-Jiguang Zhu, Meenakshi B. Bhattacharjee, Georgene W. Hergenroeder

**Affiliations:** ^1^ The Vivian L. Smith Department of Neurosurgery, McGovern Medical School at The University of Texas Health Science Center at Houston, Houston, TX, United States; ^2^ Department of Pathology & Laboratory Medicine, McGovern Medical School at The University of Texas Health Science Center at Houston, Houston, TX, United States

**Keywords:** diffuse midline glioma (DMG), diffuse midline glioma H3 K27-altered, adult DMG, H3 K27, H3K27M mutation

## Abstract

H3 K27-Altered Diffuse Midline Gliomas are commonly found in children and adolescents in midline locations such as the thalamus, brain stem, and spinal cord. It is rare for these tumors to affect adults and to occur in locations like the lateral ventricles. Despite aggressive treatment methodologies, there is no cure for this disease. The median survival is between 8-12 months. A 24-year-old white male presented to the emergency department due to severe headache refractory to pain medications with a 2-month history of progressive headaches and eventual memory problems. Computed tomography (CT) and magnetic resonance imaging (MRI) showed an intraventricular enhancing mass and hydrocephalus. The final diagnosis was an intraventricular H3 K27-Altered Diffuse Midline Glioma. The patient underwent two craniotomies, one laser interstitial thermal ablation (LITT), chemoradiotherapy, and bevacizumab and ONC206, through compassionate use. Despite a reduction in the tumor size, it continued to spread to other brain areas, leading to further complications and, eventually, his death, 10 months after initial diagnosis. From review of the literature, 21 cases were identified, and the median age was 24. Their median survival is 10.5 months (ranges 1 - 24 months). This case report presents the clinical, radiological, pathological, and molecular characteristics of a 24-year-old white man diagnosed with a ventricular H3 K27-Altered diffuse midline glioma, highlighting the rare presentation, management, and outcomes.

## Introduction

Diffuse midline gliomas (DMGs) characterized by the histone H3 K27M mutation are rare and aggressive high-grade tumors predominantly affecting children ages 5-10 years (1). The midline location defines this tumor type, diffuse growth pattern/infiltrating, and lysine-to-methionine substitution at position 27 on the H3 histone genes (2). This tumor type was first recognized in the 2016 World Health Organization (WHO) classification of central nervous system (CNS) tumors as DMG H3 K27M mutant. In 2021, WHO CNS tumor terminology changed to DMG H3 K27-altered to include subtypes of DMG with alternative mechanisms for the loss of H3K27 methylation, such as EGFR mutant DMG or EZH inhibitory protein overexpression DMG. This classification is categorized as “pediatric-type diffuse midline glioma” and is subdivided into 4 subtypes (DMG H3 K27-altered; diffuse hemispheric glioma, H3 G34-mutant; diffuse pediatric-type high-grade glioma H3-wildtype and IDH-wildtype; and infant-type hemispheric glioma), each of which possess characteristic molecular profiles (3). DMGs commonly arise in the thalamus, brainstem, and spinal cord—regions critical for vital functions—making these tumors particularly challenging to treat. These tumors are typically diagnosed in children and are associated with a very poor prognosis. The 5-year survival rate for patients with DMGs is less than 1%, and the median overall survival ranges from eight to twelve months (4).

Ventricular tumors are also rare and represent 0.8-1.6% of intracranial tumors, but tend to be benign, such as central neurocytomas, choroid plexus papillomas or carcinomas, astrocytomas, meningiomas, ependymomas, colloid cysts, or craniopharyngiomas. This case report describes the clinical, radiological, pathological, and molecular characteristics of a 24-year-old white male diagnosed with a ventricular H3 K27-altered diffuse midline glioma, highlighting the challenges and complexities of management.

## Case description

A 24-year-old white male firefighter with a history of asthma and attention deficit hyperactivity disorder (ADHD) presented with a 2-month history of nonspecific memory problems. He was described by family members as exhibiting forgetfulness of events and tasks. Developed progressive headaches that were alleviated by lying down and taking NASID medications with limited benefit. These headaches were subsequently accompanied by photophobia, phonophobia, nausea, and vomiting.

**Figure 1:**
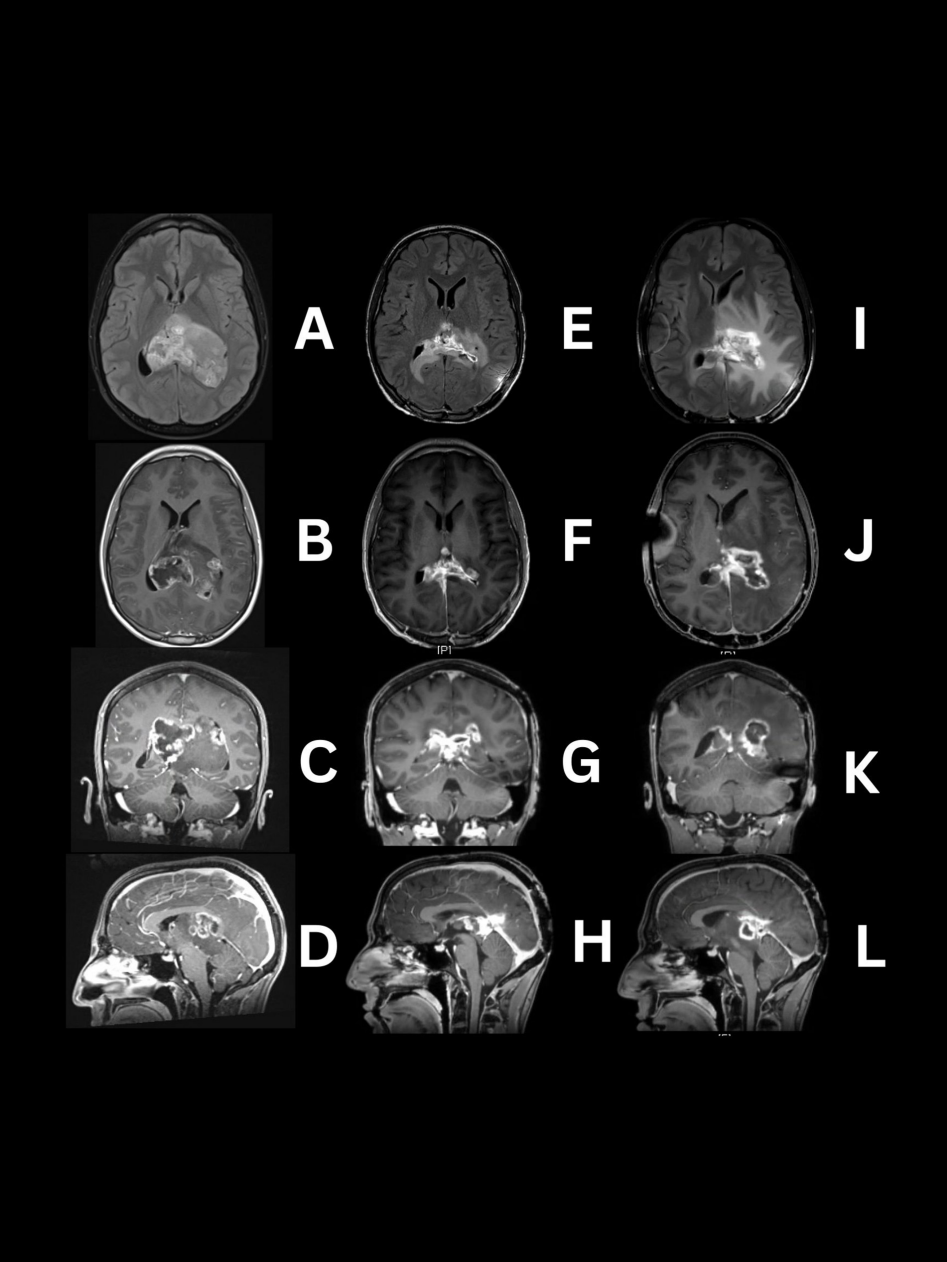
MRI images demonstrating serial progression of H3 K27-altered diffuse midline glioma. Preoperative **(A–D)** tumor located in lateral ventricles predominantly solid with patchy areas showing high intensity and central necrosis on the right side on T2/FLAIR and T1W post contrast. Day 104 post operation and chemoradiotherapy **(E–H)** reduced intraventricular tumor with periventricular FLAIR signal abnormality and contrast enhancement extending to the surrounding structures in T1W post-contrast images. Day 199 post-final treatment, **(I–L)** Progression of FLAIR signal (which is most likely a mixture of tumor infiltration with edema and radiation change) extending to basal ganglia and parietal lobes, with enhancement and necrosis of the intraventricular tumor and adjacent structures.

He presented to the emergency department due to a severe prolonged headache refractory to usual treatment, which led to a CT scan showing an intraventricular mass. A magnetic resonance image (MRI) of the brain with and without contrast revealed an irregular 6.4x7x4.5 cm (AP x Lat x CC) heterogeneous mass in the lateral ventricles appearing to be coming from the pineal region. This image did not identify involvement of midline structures like the thalamus. It occupied both lateral ventricles causing an 8 mm right midline shift (**Figures 1A–D**). The mass exhibited aggressive features, including restricted diffusion, necrosis, and heterogeneous contrast enhancement (**Figures 1B, C**) (5).

The following day, the patient underwent a left parietal craniotomy with a transparietal approach for mass resection and ventriculoperitoneal shunt (VPS) placement. The tumor location, firm and rubbery consistency, and similarity to adjacent brain tissue necessitated an initial partial resection. A postoperative MRI four days later showed expected surgical changes with a mass reduction to approximately 4.4x7x3.7 cm (AP x Lat x CC). Five days after the initial surgery, he underwent a second craniotomy with an interhemispheric approach focusing on the right lateral ventricle for further mass resection. MRI performed one day after the second surgery showed expected surgical changes and residual left intraventricular tumor of approximately 1.8x1.8x0.7cm with post-surgical periventricular enhancement of the thalamus.

**Figure 2:**
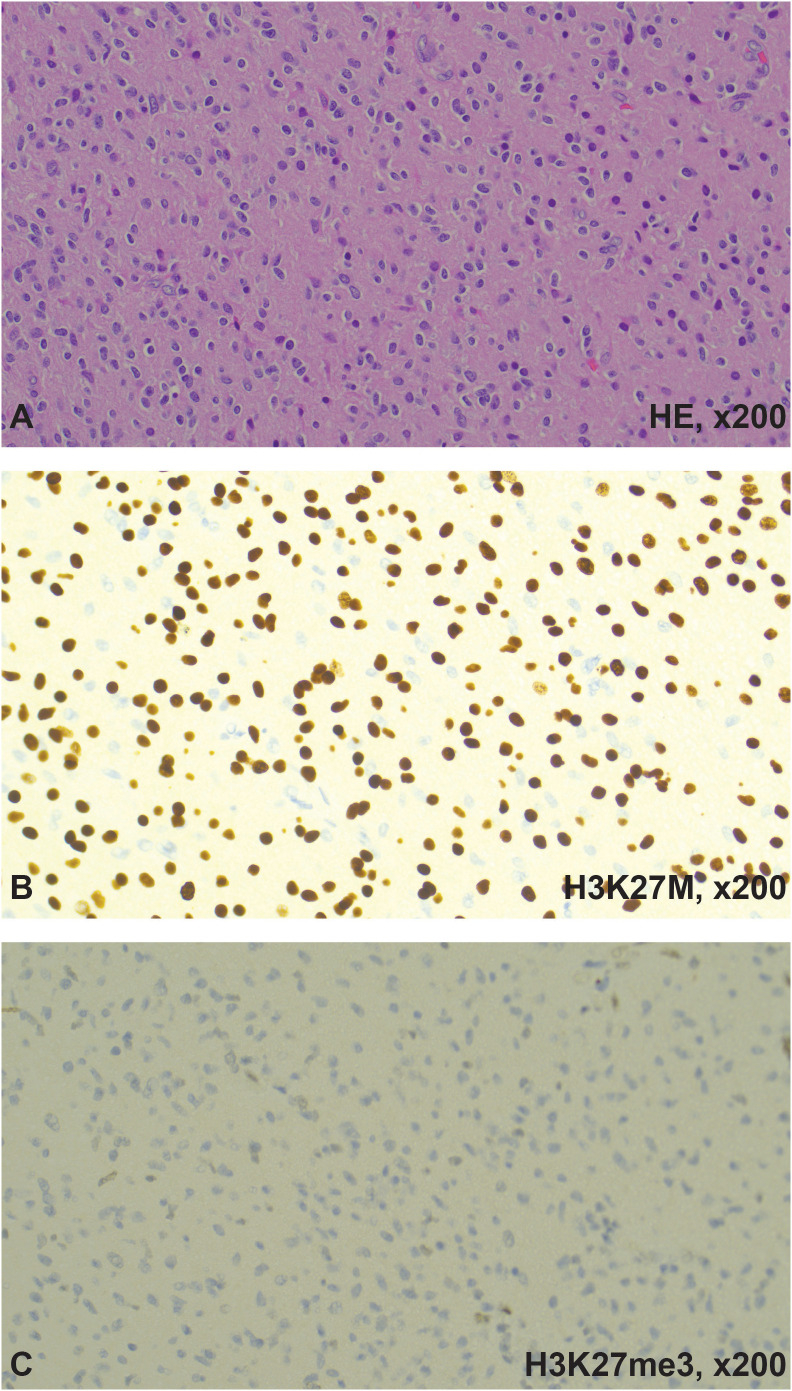
H&E and immunohistochemistry results. **(A)** Hematoxylin and Eosin (H&E) staining showed monomorphic to pleomorphic glial cells with high mitotic activity. **(B)** H3 K27M showed positive nuclear staining. **(C)** H3K27me3 showed loss of nuclear stain. The final diagnosis was DMG, H3 K27-altered, CNS WHO grade-4. Images were captured with the Leica Thunder imaging system (Danaher, Washington, DC).

The pathology report diagnosed a DMG H3 K27-Altered CNS WHO grade 4 with positive immunohistochemistry for H3K27M mutant nuclear expression, loss of H3K27me3 nuclear expression, (**Figures 2A–C**), weak to moderate nuclear expression of p53, strong expression of EGFR, and a Ki67 labeling index of 30-40%. The tumor was negative for IDH1 mutant protein expression and loss of ATRX expression. The Next Generation Sequencing (NGS) showed gene H3F3A K28M mutation, PTEN G132V – subclonal, RAD51B loss on exon 8, TSC2 E1344del, ATRX splice site 6849 + 2T>C.

Following discharge, the patient continued to experience memory difficulties and newly developed right side homonymous hemianopsia. On day 30, he began chemoradiotherapy, receiving fractionated external beam radiotherapy (54-60 Gy in 30 fractions) with concurrent temozolomide at 75 mg/m2 x 42 days. Throughout therapy, he experienced ongoing attention and memory difficulties, further visual field reduction at his right side, and seizures due to missed doses of anti-seizure medication. He completed chemoradiotherapy on Day 69.

On day 105, MRI evaluation revealed new enhancement within the splenium, left thalamus, and 3^rd^ ventricle with dimensions of 2.6x3.2x2.2 cm, raising questions about regrowth versus radiation changes (**Figures 1E–H**). The treatment approach included laser interstitial thermal ablation (LITT) through the parietal lobes with intraoperative fluoroscopy for precise targeting and ventriculoperitoneal shunt (VPS) placement. The patient was subsequently treated under a compassionate use regimen with ONC206, a more potent analogue of ONC201, a selective dopamine receptor D2 (DRD2) antagonist and mitochondrial protease ClpP agonist, at 120mg once per week, oral, for approximately 3 months.

On Day 189, his VPS malfunctioned due to blockage causing gait instability and right hemiparesis, requiring the placement of new bilateral ventriculoperitoneal shunts. He began bevacizumab therapy at a dose of 10 mg/kg IV, (without 600mg) IV, every 14 days as a salvage therapy, experiencing side effects, such as nausea, vomiting, and asthenia. The last MRI, Day 199 showed a progression in FLAIR signal in basal ganglia and parietal lobes, most likely due to tumor infiltration, radiation changes with edema or likely a mixture all three components (**Figures 1I–L**).

**Figure 3:**
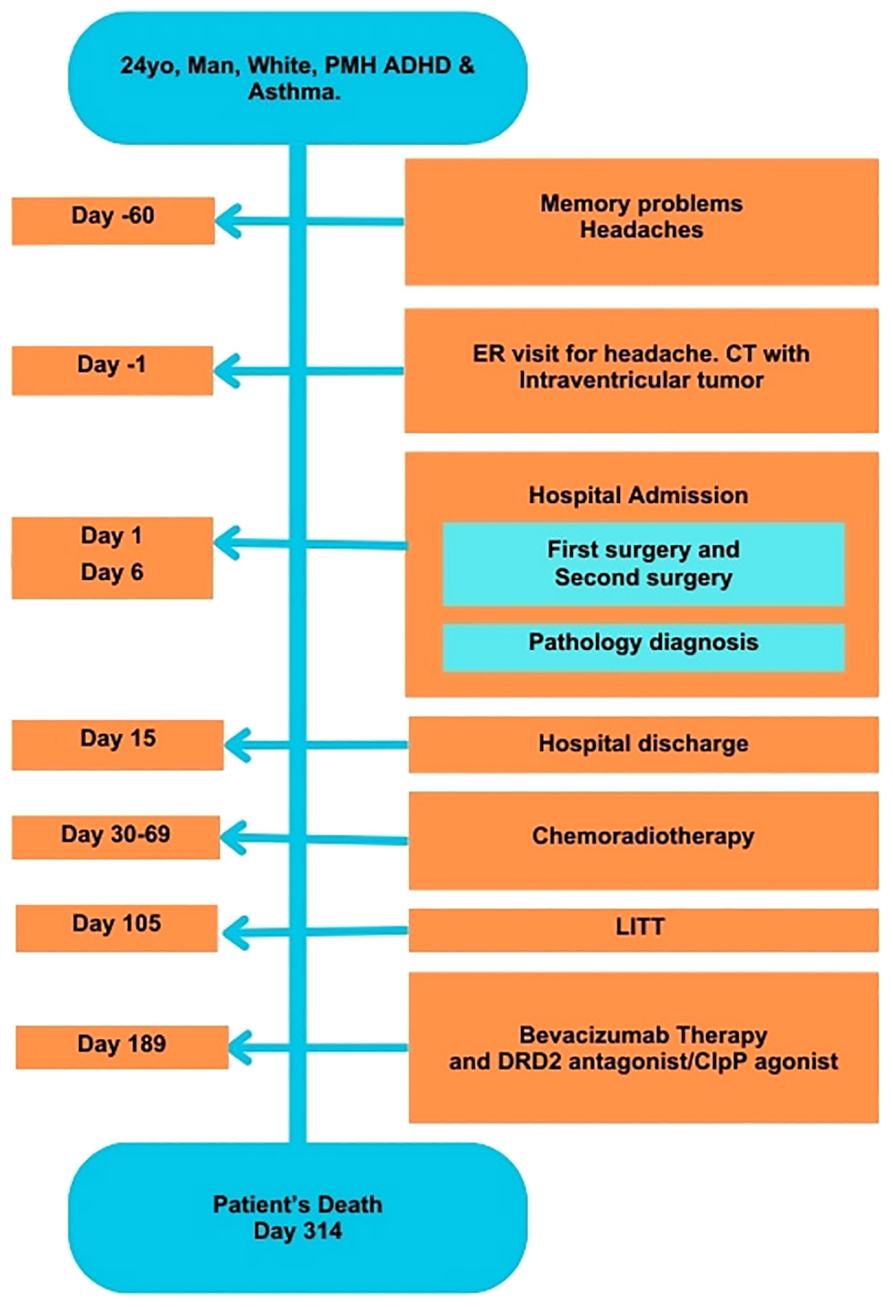
Timeline of development of symptoms and tumor treatments. ADHD, Attention Deficit Hyperactivity Disorder; ER, Emergency Room; CT, Computed tomography; LITT, Laser Interstitial Thermal ablation; PMH, Past Medical History.

On Day 314, the patient’s condition deteriorated, leading to hospitalization. Despite intensive treatment efforts, he ultimately succumbed to his illness on the same day (**Figure 3**).

## Discussion

Diffuse Midline Gliomas (DMGs) with the H3K27M mutation predominantly affect midline structures, such as the thalamus, brainstem, and spinal cord in children and adolescents. In adults, these tumors more commonly involve the diencephalic region, particularly the thalamus, with a mean age of onset around 42 years (1). This case report highlights the aggressive nature and complex management of an H3 K27-altered DMG in an adult with an unusual ventricular location, presenting significant clinical challenges.

Intraventricular tumors are rare and represent 0.8-1.6% of intracranial tumors. Intraventricular tumors are benign and are more common in childhood than adulthood. Some examples of these tumors are neoplasm of choroid plexus, ventricular wall and septum pellucid, and secondary malignant intraventricular tumors like glioblastoma multiforme. The most common clinical presentation is secondary to high ventricular pressure.

**Table 1** presents a comparative analysis of 22 reported DMG cases with ventricular involvement. Most of these cases involve patients of Asian descent, suggesting a possible racial predisposition for ventricular involvement. The median age was 24, with a median survival of 10.5 months (1-24 months).

**Table 1:** Comparison between Intraventricular DMG H3 K27-Altered cases.

Summary of Published Cases with Intraventricular DMG, H3 K27-Altered							
Reference	Age (years)	Sex	Race	Immunophenotype	No. of DMG H3 K27-Altered	Ventricle	Median survival (months)
Wang et al., 2018 (6).	Mean ± SD: 40.63 ± 21.82	Not Described	Asian	Not Described	3	Lateral	12.8
Luo et al., 2020 (7).	38	Male	Asian	GFAP, Olig2, Ki67 (+2%)	1	Lateral	24
Zhao et al., 2022 (8).	14	Female	Asian	GFAP, Olig2, S100	1	Lateral	1
Zheng et al., 2022 (9)	Median 24, Range 3 - 71	ND	Asian	Not Described	16	Not Described	10.5
Presenting case	24	Male	White	EGFR, P53, Ki67 (+30-40%), BRAF (V600E) negative	1	Lateral	10.5

EGFR, Epidermal growth factor receptor; GFAP, Glial fibrillary acidic protein; Ki67, Ki67 protein; Olig2, Oligodendrocyte transcription factor; S100, S100 protein; SD, Standard Deviation.

Clinical presentation varies depending on the anatomical area affected. In this case, the intraventricular mass caused headaches, attention, and memory problems for at least two months. These nonspecific symptoms, likely indicative of increased intracranial pressure, may have delayed diagnosis. However, given the aggressive nature of these tumors and the lack of highly effective treatments, it is unclear if an earlier diagnosis would have significantly altered the outcome.

The patient underwent two intraventricular surgeries, one-time LITT, fractionated external beam radiation (30 fractions) in combination with daily temozolomide, 3 sessions of monoclonal antibody therapy with bevacizumab at 10mg/kg every 14 days, and ONC206 drug through compassionate use. These therapies were based on the ASCO-SNO guidelines for diffuse astrocytic and oligodendroglial tumors in adults, encouraging radiotherapy and enrollment in clinical trials with this alteration (10).

The H3K27M mutation in DMG tumors is currently the primary negative prognostic factor in both adults and children. However, differentiation based on other genetic alterations could provide valuable targets for therapy, prognostication, and risk assessment. DMG tumors with H3K27M mutations in the midline region can also exhibit alterations, such as IDH negativity, ATRX loss, CDK2A deletion, TP53 overexpression, EGFR expression, and MGMT promoter methylation (11, 12).

In this case, the tumor exhibited the classic H3K27M protein expression alongside ATRX loss, moderate TP53 expression, EGFR overexpression by IHC, and H3F3A K28M mutation along with PTEN G132V – subclonal, RAD51B loss on exon 8, TSC2 E1344del, ATRX splice site 6849 + 2T>C by next generation sequencing (Foundation Medicine, Cambridge, MA). Immunohistochemical markers commonly observed in intraventricular DMG tumor case reports, were GFAP, Olig2, Ki67, and S100, were also expressed (**Table 1**).

Despite aggressive treatment, the primary ventricular tumor reduced in size by nearly 70%, yet continued to proliferate into adjacent areas like the thalamus and splenium. This progression might be linked to the tumor’s immunophenotype, characterized by EGFR overexpression, loss of H3 K27 trimethylation, a high Ki67 proliferation index (30-40%), and weak P53 expression (13).

EGFR overexpression and loss of H3 K27 trimethylation are associated with increased migratory potential and greater propensity for thalamic invasion. Furthermore, TP53 loss, a common alteration in DMGs, is known to promote tumor self-renewal, induce epigenetic dysregulation, and confer resistance to radiotherapy (12, 13).

The molecular profile of this tumor likely played a critical role in its development, migration, and response or lack of response to treatment. Interestingly, the immunophenotype in this case differed from previously reported intraventricular DMG cases, aligning more closely with diffuse midline gliomas originating in the thalamus. This unique molecular profile may have facilitated the tumor’s expansion from the ventricles into adjacent structures, including the thalamus, splenium, and third ventricle, contributing to its aggressive progression and resistance to conventional therapies.

## Conclusion

The classification of DMG H3 K27-altered tumors was first designated in 2016 and was updated in 2021 to incorporate alternative mechanisms of H3K27 methylation loss. As such, documenting and analyzing atypical cases is vital to improving our understanding of this complex disease. These tumors are more frequently seen in children and primarily affect deep midline structures. Intracranial intraventricular tumors are rare, comprising less than 1.6% of all tumors, and typically have favorable outcomes when treated. However, this case presents a difficult-to-treat tumor with an unusual growth pattern from the ventricle to diencephalic structures.

Cases like the one presented here offer an opportunity to explore whether delayed diagnoses in adults are due to different growth rates, whether the tumor phenotype varies across age groups, or if molecular markers can predict tumor progression or indicate epigenetic alterations. Current treatment protocols are not specifically designed to target this mutation, leading to highly variable prognoses. Unfortunately, no single factor has yet been definitively identified as having a significant impact on outcomes, highlighting the ongoing need for research and the development of more effective, mutation based targeted therapies.

## Data Availability

The original contributions presented in the study are included in the article. Further inquiries can be directed to the corresponding author.
